# Abnormal VLCADD newborn screening resembling MADD in four neonates with decreased riboflavin levels and VLCAD activity

**DOI:** 10.1002/jmd2.12223

**Published:** 2021-05-07

**Authors:** Marne C. Hagemeijer, Esmee Oussoren, George J. G. Ruijter, Willem Onkenhout, Hidde H. Huidekoper, Merel S. Ebberink, Hans R. Waterham, Sacha Ferdinandusse, Maaike C. de Vries, Marleen C. D. G. Huigen, Leo A. J. Kluijtmans, Karlien L. M. Coene, Henk J Blom

**Affiliations:** ^1^ Center for Lysosomal and Metabolic Diseases, Department of Clinical Genetics Erasmus University Medical Center Rotterdam The Netherlands; ^2^ Center for Lysosomal and Metabolic Diseases, Department of Pediatrics Erasmus University Medical Center Rotterdam The Netherlands; ^3^ Laboratory Genetic Metabolic Diseases, Department of Clinical Chemistry, Amsterdam Gastroenterology Endocrinology Metabolism Amsterdam UMC, University of Amsterdam Amsterdam The Netherlands; ^4^ Department of Laboratory Medicine, Translational Metabolic Laboratory (TML) Radboud University Medical Center Nijmegen The Netherlands

**Keywords:** MADD, newborn screening, riboflavin deficiency, VLCADD

## Abstract

Early detection of congenital disorders by newborn screening (NBS) programs is essential to prevent or limit disease manifestation in affected neonates. These programs balance between the detection of the highest number of true cases and the lowest number of false‐positives. In this case report, we describe four unrelated cases with a false‐positive NBS result for very‐long‐chain acyl‐CoA dehydrogenase deficiency (VLCADD). Three neonates presented with decreased but not deficient VLCAD enzyme activity and two of them carried a single heterozygous *ACADVL* c.1844G>A mutation. Initial biochemical investigations after positive NBS referral in these infants revealed acylcarnitine and organic acid profiles resembling those seen in multiple acyl‐CoA dehydrogenase deficiency (MADD). Genetic analysis did not reveal any pathogenic mutations in the genes encoding the electron transfer flavoprotein (ETF alpha and beta subunits) nor in ETF dehydrogenase. Subsequent further diagnostics revealed decreased levels of riboflavin in the newborns and oral riboflavin administration normalized the MADD‐like biochemical profiles. During pregnancy, the mothers followed a vegan, vegetarian or lactose‐free diet which probably caused alimentary riboflavin deficiency in the neonates. This report demonstrates that a secondary (alimentary) maternal riboflavin deficiency in combination with reduced VLCAD activity in the newborns can result in an abnormal VLCADD/MADD acylcarnitine profile and can cause false‐positive NBS. We hypothesize that maternal riboflavin deficiency contributed to the false‐positive VLCADD neonatal screening results.

**Synopsis**

Maternal riboflavin deficiency in combination with decreased VLCAD activity can result in false‐positive VLCADD neonatal screening results with MADD‐like biochemistry.

## 1 INTRODUCTION

Mitochondrial fatty acid beta‐oxidation (FAO) is one of the main metabolic pathways needed to maintain proper energy homeostasis, in particular during prolonged periods of fasting. Fatty acids are utilized to generate acetyl‐coenzyme A (Ac‐CoA) that is (i) transferred to the tricarboxylic acid cycle or (ii) converted into ketone bodies in the liver to serve as metabolic fuel throughout the body, including the brain.^1^, ^2^

Dutch newborns are currently officially tested for a total of 22 inherited disorders in the national newborn blood spot screening (NBS) program (https://www.rivm.nl/en/heel-prick/clinical-picture) with the majority being inborn errors of metabolism (IEMs). Five of these diseases are FAO defects, which are detected by quantification of acylcarnitine species in dried blood spots (DBS) obtained 3 to 7 days after birth (https://www.pns.nl/node/3471). Abnormal acylcarnitine profiles obtained during newborn screening requires follow‐up testing at specialized academic medical centers to confirm (or exclude) the abnormal screening result, to identify the exact metabolic defect and for an early start of adequate treatment if a metabolic disorder is confirmed.

Here, we report four cases identified after a positive newborn screening for very‐long‐chain acyl‐CoA dehydrogenase deficiency (VLCADD; OMIM #201475) based on an increased C14:1/C2 acylcarnitine ratio. Further biochemical testing in plasma, however, revealed acylcarnitine profiles resembling multiple acyl‐CoA dehydrogenase deficiency (MADD; OMIM #231680). Additional biochemical and genetic studies in these neonates showed (i) decreased/reduced VLCAD enzyme activity in three out of four neonates, (ii) the presence of a heterozygous *ACADVL* c.1844G>A mutation in two of the infants (carrier status), and (iii) decreased levels of riboflavin in all the neonates. We hypothesize that maternal riboflavin deficiency induced riboflavin deficiency in these infants, which in combination with decreased VLCAD activity, resulted in false‐positive VLCADD neonatal screening.

## 2 METHODS

### 2.1 Biochemical analyses

In laboratories specialized in the diagnostics of IEM, urine organic acids were analyzed by gas chromatography/mass spectrometry (GC/MS) and plasma acylcarnitine profiling was performed by liquid chromatography (LC)‐MS/MS. Plasma flavin concentrations were analyzed by high‐performance liquid chromatography‐fluorescence detection as previously published with minor modifications.^3^ All biochemical analyses were performed according to well‐established diagnostic protocols under ISO 15189 accreditation.

### 2.2 VLCAD enzyme activity measurements

VLCAD enzyme activity was quantitatively assessed in patient‐derived lymphocytes as previously published.^4^

### 2.3 Genetic studies

Our genetic studies were performed using Sanger‐sequencing or whole‐exome sequencing. These approaches only allow for analysis of the protein‐coding regions of the investigated genes and as such deletions or intronic mutations are not detected by these techniques. The following genes were analyzed: (i) VLCADD: very‐long‐chain acyl‐CoA dehydrogenase (*ACADVL*; OMIM *609575); (ii) MADD: electron transfer flavoprotein subunit alpha (*ETFA*; OMIM *608053), subunit beta (*ETFB*; OMIM *130410), and electron transfer flavoprotein dehydrogenase (*ETFDH*; OMIM *231675); (iii) riboflavin transport: solute carrier family 52 members 1‐3 (*SLC52A1‐3*; OMIM *607883, *607882, and *613350); (iv) FAD synthesis: flavin adenine dinucleotide synthetase 1 (*FLAD1*; OMIM *610595); and (v) FAD transport: solute carrier family 25 member 32 (*SLC25A32*; OMIM *138480).

## 3 RESULTS

Four unrelated newborns were referred to an academic medical center because of a positive newborn screening for VLCADD. All newborns were born at term, healthy, and after birth exclusively breastfed. An overview of the newborn screening results is provided in Table 1. All four of the investigated bloodspots showed normal levels of free carnitine and an elevated C14:1/C2 acylcarnitine ratio (≥0.023). Subject 2 had slightly increased levels of tetradecenoylcarnitine (C14:1‐carnitine). No other aberrant acylcarnitine species (i.e., C5‐, C8‐, and C10‐carnitine) were detected in the bloodspots of all of the neonates (data not shown).

**TABLE 1:** Overview newborn screening results in DBS

	ID 1	ID 2	ID 3	ID 4	Out‐of‐range values^a^
Free carnitine	13.9	22.4	8.7	15.9	≤5 μmol/L
C14:1	0.21	**0.61**	0.30	0.56	≥0.60 μmol/L
Ratio C14:1/C2	**0.023**	**0.038**	**0.029**	**0.033**	≥0.023

*Note*: Aberrant values are indicated in bold.

Abbreviations: *C14:1*, tetradecenoylcarnitine; *C2*, acetylcarnitine.

^a^ These are the Dutch NBS cutoff values of which only one of the two VLCADD markers (either C14:1 or the C14:1/C2 ratio) needs to be abnormal for referral to academic medical centers for additional confirmatory diagnostic testing. All of the DBS samples were obtained 4 days after birth.

After referral, additional confirmatory diagnostic testing of acylcarnitines in plasma and organic acids in urine was performed in each newborn (Table 2). Normal free carnitine levels, together with mildly elevated concentrations of C14:1‐carnitine, were detected in all plasma samples. The C14:1/C2 acylcarnitine ratio was only slightly elevated in subjects 1 and 4. Remarkably, the acylcarnitine profiles showed additional increased acylcarnitine concentrations in all samples, including short‐ (C4, C5), medium‐ (C6, C8, and C10), and long‐ (C12, C14) chain acylcarnitines (Table 2). This pattern of multiple elevated acylcarnitines is not associated with VLCADD but rather with decreased activities of FAD‐dependent dehydrogenases as seen in multiple acyl‐CoA dehydrogenase deficiency (MADD, or glutaric aciduria type II). In line with these results, organic acids analyses in urine of subjects 1, 2, and 4 showed characteristic MADD metabolites, including the dicarboxylic acids ethylmalonic acid, adipic acid and suberic acid, the glycine conjugates hexanoylglycine, isovalerylglycine and suberylglycine, and 2‐hydroxyglutaric acid (Table 2).

**TABLE 2:** Overview biochemical screening results

Acylcarnitine analysis (μmol/L)	ID1 (*d6*)	ID2 (*d7*)	ID3 (*d8*)	ID4 (*d9*)
C0	23 (22.3‐54.8)	24 (22.3‐54.8)	**15** (22.3‐55)	29 (20‐55)
C2	4.4 (3.4‐13)	7.5 (3.4‐13)	6.0 (3.4‐13)	5.5 (2‐9.04)
C3	0.28 (0.14‐0.94)	0.88 (0.14‐0.94)	0.24 (0.14‐0.94)	0.74 (0.23‐0.92)
C4	0.32 (0.07‐0.58)	**0.87** (0.07‐0.58)	0.33 (0.07‐0.58)	**0.63** (0‐0.55)
C5	0.23 (0.07‐0.22)	**0.85** (0.07‐0.22)	0.10 (0.04‐0.22)	**0.78** (0‐0.24)
C6	**0.16** (0‐0.12)	**0.27** (0‐0.12)	0.10 (0.02‐0.12)	**0.20** (0‐0.12)
C8	**0.28** (0.04‐0.22)	**0.32** (0.04‐0.22)	**0.37** (0.04‐0.22)	**0.30** (0‐0.22)
C10	**0.41** (0‐0.3)	**0.45** (0‐0.3)	**0.51** (0.04‐0.30)	**0.53** (0‐0.31)
C12	**0.25** (0‐0.14)	**0.33** (0‐0.14)	**0.31** (0.04‐0.14)	**0.33** (0‐0.14)
C14:1	**0.26** (0.02‐0.18)	**0.21** (0.02‐0.18)	**0.24** (0.02‐0.18)	**0.38** (0‐0.17)
C14	0.13 (0‐0.16)	0.17 (0‐0.16)	0.09 (0‐0.08)	**0.15** (0‐0.13)
C16	0.19 (0.06‐0.24)	0.14 (0.06‐0.24)	0.17 (0.06‐0.24)	0.15 (0.03‐0.23)
C18	0.06 (0.02‐0.1)	0.04 (0.02‐0.1)	0.03 (0.02‐0.10)	0.05 (0‐0.09)
Ratio C14:1/C2	**0.06** (<0.05)	0.03 (<0.05)	0.04 (<0.05)	**0.07** (<0.06)
**Organic acid analysis (mmol/mol creatinine)**	**ID1 (*d10*)**	**ID2 (*d9*)**	**ID3 (*d12*)**	**ID4 (*d11*)**
2‐hydroxyglutaric acid	**165** (0‐50)	**123** (0‐50)	31 (0‐50)	↑^#^
3‐hydroxyglutaric acid	**7** (0‐5)	**9** (0‐5)	n.d.	‐
Acetoacetate	4 (0‐5)	n.d.	**8** (0‐5)	Normal^#^
Adipic acid	**52** (0‐20)	8 (0‐20)	18 (0‐20)	**143** (0‐30)
Ethylmalonic acid	14 (0‐30)	23 (0‐30)	7 (0‐30)	**26** (0‐20)
Glutaric acid	5 (0‐5)	**7** (0‐5)	1 (0‐5)	14 (0‐30)
Hexanoylglycine	**4** (0‐1)	**4** (0‐1)	n.d.	n.d.
Isovalerylglycine	**2** (0‐1)	**12** (0‐1)	n.d.	n.d.
Suberic acid	**12** (0‐10)	3 (0‐10)	6 (0‐10)	**28** (0‐15)
Suberylglycine	n.d.	n.d.	n.d.	n.d.
**Enzyme activity [nmol/(min.mg protein)]**	**ID1 (*d10*)**	**ID2 (*d7*)**	**ID3 (*d8*)**	**ID4 (*d9*)**
VLCAD	**1.07** (2.15‐3.79)	**1.62** (2.15‐3.79)	3.14 (2.15‐3.79)	**1.97** (2.15‐3.79)
**Genotype**	**ID1**	**ID2**	**ID3**	**ID4**
*ACADVL* c.1844G>A	Heterozygous	Heterozygous	‐	n.d.
**Flavines (nmol/L)**	**ID1 (d10)**	**ID2 (*d7*)**	**ID3 (*d8*)**	**ID4 *(d9)***
FAD	**34** (46.0‐114.0)	46.1 (46.0‐114.0)	60.0 (46.0‐114.0)	**38.4** (46.0‐114.0)
FMN	**2.2** (2.8‐21.4)	**0.2** (2.8‐21.4)	3.6 (2.8‐21.4)	**1.1** (2.8‐21.4)
Riboflavin	**2.9** (3.9‐49.0)	**2.8** (3.9‐49.0)	**2.4** (3.9‐49.0)	**2.9** (3.9‐49.0)
**Methylmalonic acid**	**ID1**	**ID2**	**ID3**	**ID4**
Plasma (μmol/L)	0.19 (0‐0.32) (*d131*)	**6.68** (0‐0.32) (*d70*)	n.d.	**7.1** (0‐0.31) (*d22*)
Urine (mmol/mol creatinine)	4 (0‐20) (*d10*)	19 (0‐20) (*d9*)	1 (0‐20) (*d12*)	**86** (URL 10) (*d11*)

*Notes*: The values depicted in the table were measured in the first samples obtained after the newborn screening results. The center‐specific reference ranges are indicated in brackets; # not quantitatively determined. Aberrant values are indicated in bold.

Abbreviations: *C0*, free carnitine; *C2*, acetylcarnitine; *C3*, propionylcarnitine; *C4*, butyrylcarnitine; *C5*, isovaleryl‐/methylbutyrylcarnitine; *C6*, hexanoylcarnitine; *C8*, octanoylcarnitine; *C10*, decanoylcarnitine; *C12*, dodecanoylcarnitine; *C14:1*, tetradecenoylcarnitine; *C14*, tetradecanoylcarnitine; *C16*, hexadecanoylcarnitine; *C18*, octadecanoylcarnitine; *d*, days after birth; FAD, flavin adenine dinucleotide; FMN, flavin mononucleotide; n.d., not detected; URL, upper range limit; *VLCAD*, very‐long chain acyl‐CoA dehydrogenase.

VLCAD enzyme activity in lymphocytes was decreased for subjects 1, 2, and 4, but higher than normally found in patients with a genetically confirmed VLCADD. VLCAD enzyme activity was normal in lymphocytes of subject 3 (Table 2). The *ACADVL* gene of subjects 1, 2, and 4 was sequenced and revealed a single heterozygous c.1844G>A (p.Arg615Gln) *ACADVL* mutation in subjects 1 and 2, which has been described before.^5^, ^6^, ^7^, ^8^, ^9^ No other mutations in the *ACADVL* gene of these patients were found. This finding was in agreement with the decreased, but not deficient VLCAD enzyme activity. Sequencing of the coding region of the *ACADVL* gene of subject 4 did not reveal any pathogenic variant. DNA analysis of the *ACADVL* gene in subject 3 was not performed as this subject exhibited normal VLCAD enzyme activity. Subsequently, the genes involved in MADD, i.e. the electron transfer flavoprotein (*ETFA* and *ETFB*) and ETF‐ubiquinone oxidoreductase (*ETFDH*), were analyzed in all four patients, but no pathogenic mutations were detected in any of the subjects.

FAD is a main co‐factor for correct functioning and linking of acyl‐CoA dehydrogenases to the electron respiratory chain.^1^, ^2^ Since defects in the transport or metabolism of riboflavin, the precursor of the biologically active cofactor flavin adenine dinucleotide (FAD), can also cause MADD(‐like) metabolite abnormalities,^10^, ^11^, ^12^, ^13^, ^14^, ^15^, ^16^, ^17^ we investigated the riboflavin status of these subjects. Plasma concentrations of FAD and the precursors flavin mononucleotide (FMN) and riboflavin were determined and were all decreased for subjects 1, 2, and 4, except for FAD in subject 2. Subject 3 only demonstrated decreased levels of riboflavin with normal plasma levels of FMN and FAD (Table 2). Despite the affected riboflavin status of these individuals, mutational analysis of the genes involved in riboflavin transport (*SLC52A1/SLC52A2/SLC52A3*) did not reveal any pathogenic variants. Additional investigation of genes involved in the synthesis (*FLAD1*) or transport (*SLC25A32*) of FAD of subject 4 did not reveal any abnormalities as well.

Treatment of these infants with oral riboflavin (subjects ID1, ID2, and ID3 received 60 mg/day and subject ID4 50 mg/day) resulted in normalization of the acylcarnitine and organic acid profiles, suggesting that the MADD‐like metabolic abnormalities were due to riboflavin deficiency (*data not shown*). In all subjects the normal biochemistry persisted even after ending the riboflavin treatment, which implies an acquired riboflavin deficiency via their mothers. Taking a careful medical history of the mothers revealed that (during pregnancy) they either had a lactose‐free, a vegan, a vegetarian diet (cases 1, 2, and 4), or an imbalanced diet (case 3), which might have resulted in the observed (alimentary) riboflavin deficiency in these infants.

## 4 DISCUSSION

Here, we report four asymptomatic neonates that presented with an abnormal NBS for VLCADD. Enzyme activity of VLCAD in lymphocytes was decreased, but not deficient, in three out of four newborns with a normal activity in one. Biochemical studies in plasma and urine showed MADD‐like metabolic profiles in all four cases. Further investigations revealed an acquired riboflavin deficiency in these newborns and subsequent treatment with riboflavin normalized the observed biochemical abnormalities.

Follow‐up testing of the neonates demonstrated characteristic plasma acylcarnitine profiles (elevated C4‐C16 acylcarnitine species) and organic acid urine patterns (e.g. 2‐hydroxyglutaric acid and specific glycine conjugates) indicative of MADD. However, genetic screening of genes directly involved in MADD (*ETFA*, *ETFB*, and *ETDFH*) in all subjects did not reveal any genetic variants that could explain these biochemical profiles.

In the Netherlands, the newborn screening cutoff values of VLCADD are C14:1 ≥ 0.60 μmol/L and C14:1/C2 ratio ≥ 0.023 in DBS.^9^ Subsequent metabolic investigations in plasma of the four newborns demonstrated only marginally aberrant C14:1/C2 ratios with decreased but not deficient VLCAD enzyme activity in lymphocytes in three out of four cases. Aberrant NBS acylcarnitine results due to *ACADVL* heterozygosity is not unusual and requires follow‐up biochemical investigations.^18^, ^19^, ^20^, ^21^ Newborns switch to anabolic conditions in the days following birth and normalization of acylcarnitine profiles in repeat samples is not uncommon.^21^, ^22^ It does, however, result in a decrease of the sensitivity of the VLCADD markers. Nonetheless, in the period between 2015 and 2019 the positive predictive value (PPV) of the VLCADD screening in the Netherlands was 33% with a sensitivity and specificity of 95.65% and 99.99%, respectively.^23^ False‐positive VLCAD diagnoses, like the ones described here, do occur but not frequently.

Genetic analysis of the *ACADVL* gene revealed that two newborns harbored a previously published heterozygous c.1844G>A (p.Arg615Gln) *ACADVL* missense mutation. This mutation has been described as a variant of uncertain significance,^24^ which is in line with the findings presented in this study. The two unrelated neonates in this case report who were heterozygous for this mutation showed residual VLCAD activity in lymphocytes.

MADD‐like biochemical profiles have also been reported for defects in riboflavin transport or metabolism.^10^, ^11^, ^12^, ^13^, ^14^, ^15^, ^16^, ^17^ Riboflavin is the precursor of FAD, which is the essential cofactor for correct acyl‐CoA dehydrogenase activity. FAD is generated from riboflavin (vitamin B_2_) via FMN by riboflavin kinase, which in turn is converted into FAD by FAD synthase. Riboflavin has to be transported from the bloodstream into cells, which is mediated by cellular riboflavin transporters.^25^ As FAD is the cofactor for VLCAD, the reduced riboflavin status as demonstrated in our patients may have contributed to the false positive newborn screening. Analysis of several of the genes involved in riboflavin transport and metabolism did not reveal any pathogenic variants which could explain the biochemical phenotype. However, as flavin levels did show (mildly) decreased levels in all infants (except for FMN and FAD in subject 3), we concluded that the observed MADD‐like biochemical phenotype was likely due to acquired riboflavin deficiency.

Riboflavin has to be obtained exclusively from the diet, especially from animal products like dairy and meat, as humans are unable to synthesize riboflavin. In pregnancy, it is passed from the mother to the child via the placenta probably using the riboflavin transporter RFVT1 (*SLC52A1*).^26^ Transient MADD‐like biochemical profiles have been reported previously in healthy infants of whom the mothers were (suspected to be) riboflavin deficient during pregnancy.^15^, ^16^, ^27^ Medical history taking of the infants' mothers described in our case report revealed that all of them either followed an imbalanced or lactose‐free diet or were (temporarily) vegan or vegetarian during pregnancy. Although the putative genes were not investigated in the mothers, we cannot rule out potential defects in FAO or riboflavin transport or metabolism. Noteworthy, the mothers showed no symptoms associated with these defects. Treatment of the newborns with oral riboflavin restored the MADD‐like biochemical profiles to normal which persisted when riboflavin supplementation was stopped. Although we do not know the flavin status of the mothers during pregnancy, we assume that the observed MADD‐like profiles were due to maternal alimentary riboflavin deficiency.

During pregnancy, the demand for vitamins such as cobalamin (vitamin B_12_) and folic acid (vitamin B_9_) is high and can potentially lead to deficiencies of these micronutrients in the mother and in the child if intake is inadequate and not properly supplemented.^28^ We observed elevated methylmalonic acid levels in plasma of subjects 2 and 4 and in urine of subject 4 (Table 2) that normalized over time, which underscores our hypothesis of an alimentary maternal deficiency of meat‐ and dairy‐derived vitamins in these infants like cobalamin and riboflavin. Our findings support the notion that vitamin supplementation should be advised to women who intend to become pregnant, especially when mothers are planning to breast feed their infants.

Taken together, these four cases demonstrate that decreased riboflavin levels can result in false‐positive VLCADD newborn screening presenting with a MADD‐like metabolic profile, which can be corrected by oral riboflavin supplementation. Although newborn screening programs are aimed at diagnosing congenital disorders incidental findings as presented in this case report provide an opportunity to detect in newborns, and subsequently their mothers, acquired vitamin deficiencies which are treatable and would otherwise have gone unnoticed.

## CONFLICT OF INTEREST

The authors declared no potential conflicts of interest.

## INFORMED CONSENT STATEMENT

All procedures followed were in accordance with the ethical standards of the responsible committee on human experimentation (institutional and national) and with the Helsinki Declaration of 1975, as revised in 2000 (5). The legal representatives of the neonates that were included in this case report provided consent for publication of the data as described in this case report.

This article does not contain any studies with human or animal subjects performed by any of the authors.
